# Sevoflurane‐induced overexpression of extrasynaptic α5‐GABA_A_R via the RhoA/ROCK2 pathway impairs cognitive function in aged mice

**DOI:** 10.1111/acel.14209

**Published:** 2024-06-02

**Authors:** Zhun Wang, Jinpeng Dong, Mengxue Zhang, Sixuan Wang, Jiangnan Wu, Shengran Wang, Yuan Luo, Yongan Wang, Yiqing Yin

**Affiliations:** ^1^ Department of Anesthesiology Tianjin Medical University Cancer Institute and Hospital, National Clinical Research Center for Cancer Tianjin China; ^2^ Key Laboratory of Cancer Prevention and Therapy Tianjin China; ^3^ Tianjin's Clinical Research Center for Cancer Tianjin China; ^4^ Department of Anesthesiology Shandong Provincial Hospital Affiliated to Shandong First Medical University Jinan China; ^5^ State Key Laboratory of Toxicology and Medical Countermeasures Academy of Military Medical Sciences Beijing China

**Keywords:** fasudil hydrochloride, perioperative neurocognitive disorder, RhoA/ROCK2, sevoflurane, α5‐GABA_A_R

## Abstract

Perioperative neurocognitive disorder (PND) is a serious neurologic complication in aged patients and might be associated with sevoflurane exposure. However, the specific pathogenesis is still unclear. The distribution of α5‐GABA_A_R, a γ‐aminobutyric acid type A receptor (GABA_A_R) subtype, at extrasynaptic sites is influenced by the anchor protein radixin, whose phosphorylation is regulated via the RhoA/ROCK2 signaling pathway and plays a crucial role in cognition. However, whether sevoflurane affects the ability of radixin phosphorylation to alter extrasynaptic receptor expression is unknown. Aged mice were exposed to sevoflurane to induce cognitive impairment. Both total proteins and membrane proteins were extracted for analysis. Cognitive function was evaluated using the Morris water maze and fear conditioning test. Western blotting was used to determine the expression of ROCK2 and the phosphorylation of radixin. Furthermore, the colocalization of p‐radixin and α5‐GABA_A_R was observed. To inhibit ROCK2 activity, either an adeno‐associated virus (AAV) or fasudil hydrochloride was administered. Aged mice treated with sevoflurane exhibited significant cognitive impairment accompanied by increased membrane expression of α5‐GABA_A_R. Moreover, the colocalization of α5‐GABA_A_R and p‐radixin increased after treatment with sevoflurane, and this change was accompanied by an increase in ROCK2 expression and radixin phosphorylation. Notably, inhibiting the RhoA/ROCK2 pathway significantly decreased the distribution of extrasynaptic α5‐GABA_A_R and improved cognitive function. Sevoflurane activates the RhoA/ROCK2 pathway and increases the phosphorylation of radixin. Excess α5‐GABA_A_R is anchored to extrasynaptic sites and impairs cognitive ability in aged mice. Fasudil hydrochloride administration improves cognitive function.

## Graphical abstract

Sevoflurane activates the RhoA/ROCK2 pathway and increases the phosphorylation of radixin. Excess α5‐GABA_A_R is anchored to extrasynaptic sites and impairs cognitive ability in aged mice. Fasudil hydrochloride administration ameliorates cognitive impairment induced by sevoflurane.

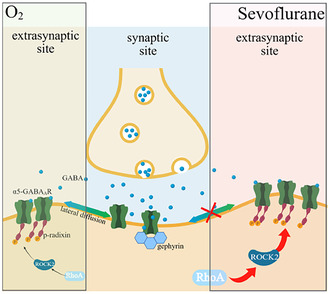

**Abbreviations**
AAV: adeno‐associated virus
Aβ: β‐amyloid protein
FCT: fear conditioning test
GABA_A_R: γ‐aminobutyric acid type A receptor
MAC: minimum alveolar concentration
MWM: Morris water maze
NKA: sodium potassium ATPase
PaCO_2_
: arterial partial pressure of carbon dioxide
PaO_2_
: arterial partial pressure of oxygen
PND: perioperative neurocognitive disorder
SaO_2_
: arterial oxygen saturation
α5‐GABA_A_R: α5 subunit‐containing GABA_A_R

## 1 INTRODUCTION

Perioperative neurocognitive disorder (PND) is a severe syndrome of the central nervous system whose symptoms include deterioration of memory, reduction of learning ability, and impairment of cognition and concentration (Li et al., 2022; Moller et al., 1998). It not only affects the quality of life of patients but also increases the burden on families and society (Kimchi et al., 2017; Schmitt et al., 2015). Although PND can occur at any age, older people are more likely to be affected (Terrando et al., 2011). With the aging of the global population, the number of elderly patients requiring surgical treatment is increasing. Despite an increasing number of investigations exploring the mechanisms underlying PND, its etiology is still unknown. Therefore, there is an urgent need to determine the pathogenesis of PND.

As one of the most commonly used volatile anesthetics in clinical practice, sevoflurane is odorless, does not irritate the airway, and is well tolerated by patients of all ages, making it widely used in many types of surgery. However, some multicenter clinical investigations have shown that individuals administered sevoflurane exhibit a significantly greater incidence of PND than those treated with propofol (Cao et al., 2023; Zhang et al., 2018). Other studies have revealed that exposure to sevoflurane may influence the expression of specific synaptic proteins and, moreover, may alter the cognitive faculties of experimental subjects, thereby corroborating the neurotoxic potential of sevoflurane (Chung et al., 2017; Guo et al., 2018; Liang et al., 2021). To determine the precise mechanism underlying the cognitive dysfunction induced by sevoflurane, an increasing number of molecular and cellular cascades, including those related to neuroinflammation, neuronal apoptosis, the accumulation of β‐amyloid protein (Aβ), and changes in synaptic plasticity, have been proposed (Chung et al., 2017; Ye et al., 2019; Zhang et al., 2020). Nonetheless, it is unclear which mechanisms play major roles in this disease.

Although the precise targets of sevoflurane remain elusive, γ‐aminobutyric acid (GABA) type A receptor (GABA_A_R) has been pinpointed as a significant point of interaction (Rudolph & Antkowiak, 2004). GABA is the major inhibitory neurotransmitter in the mammalian central nervous system, and its type A receptor is a heteropentameric ligand‐gated ion channel composed of homologous subunits. To date, a total of 19 subunits of eight major classes have been identified, namely, α1‐6, β1‐3, γ1‐3, θ, δ, ε, π, and ρ1‐3 (Engin et al., 2018). Further studies have indicated that the α5 subunit‐containing GABA_A_R (α5‐GABA_A_R) is involved in the process of memory impairment induced by anesthetic drugs (Wang, Kaneshwaran, et al., 2018; Zhao et al., 2019; Zurek et al., 2014).

The α5‐GABA_A_R is closely related to cognitive processes, not only because it is located in the hippocampus (Mohamad & Has, 2019), which is a zone closely associated with learning and memory but also because cognitive function is significantly enhanced in α5‐GABA_A_R knockout mice or after treatment with inhibitors of the α5‐GABA_A_R, such as L‐655708. Therefore, numerous scholars are focusing on the α5‐GABA_A_R as a target molecule in the study of cognition‐related diseases (Lecker et al., 2013). Saab et al. (2010) reported that tonic current in mouse brain slices was significantly enhanced after treatment with isoflurane or sevoflurane in conjunction with impaired hippocampus‐dependent cognitive function. The tonic current is mediated mainly by extrasynaptically distributed α5‐GABA_A_R. In addition, the researchers found that α5‐GABA_A_R could generate tonic current at extrasynaptic sites because of the presence of radixin, an anchor protein that is located at extrasynaptic sites. After being phosphorylated via the RhoA/ROCK2 signaling pathway, radixin can anchor α5‐GABA_A_R to the cytoskeleton, thus acting as a “bridge” (Hausrat et al., 2015; Jacob, 2019). However, there have been no studies on whether the phosphorylation of radixin is altered or whether it affects the expression of α5‐GABA_A_R after exposure to sevoflurane.

Therefore, the purpose of this study was to investigate the changes in α5‐GABA_A_R in sevoflurane‐induced cognitive dysfunction and the associated regulatory mechanism. We hypothesize that the phosphorylation of radixin is significantly elevated after sevoflurane exposure via the RhoA/ROCK2 signaling pathway. This process is believed to increase the expression of extrasynaptic α5‐GABA_A_R on the cell membrane, leading, as a consequence, to the emergence of cognitive dysfunction in aged mice.

## 2 METHODS

### 2.1 Animals

Aged C57BL/6 mice (female, 18–19 months old) and young C57BL/6 mice (female, 2–3 months old) were purchased from Wukong Biotechnology (Jiangsu, China) and SPF (Beijing) Biotechnology Co., Ltd., respectively. All animals were housed in five per cage in a controlled environment (22 ± 1°C; 50%–60% humidity; 12‐h light/dark cycle) with free access to food and water. All protocols were approved by the Animal Ethical and Welfare Committee of Tianjin Medical University Cancer Institute and Hospital (BRTF‐AE‐2023001).

### 2.2 Anesthesia

Young and aged mice were randomly divided into control (CON) and sevoflurane (SEV) groups. Mice in the sevoflurane group were placed in a transparent anesthesia induction chamber and anesthetized with sevoflurane for 2 h. Sevoflurane at a concentration of 4% was loaded into the induction chamber with 100% oxygen at a flow rate of 1 L/min. The sevoflurane concentration, end‐expiratory CO_2_ concentration in the induction chamber, heart rate, pulse oxygen saturation, and central temperature of the mice were continuously monitored to ensure the safety of the mice. A temperature‐controlled heating pad was used to maintain the body temperature of the mice throughout the administration of anesthesia. At the end of the administration of anesthesia, the mice were quickly transferred to another heating pad and allowed to awaken naturally. Throughout the duration of the procedure, the core body temperature of the mice was maintained at 37 ± 0.5°C. Mice in the control group were placed in an anesthesia induction chamber and exposed to 100% oxygen for the same duration.

### 2.3 Blood gas analysis

A separate group of mice was subjected to blood gas analysis to ensure that the mice did not develop hypoxemia during anesthesia administration. Immediately after the administration of anesthesia or oxygen, the mice in the sevoflurane group were dissected to fully expose the abdominal aorta to obtain arterial blood samples. Mice in the control group received 50 mg/kg pentobarbital sodium intraperitoneally for anesthesia, and after their rollover reflex disappeared, the abdominal aorta was exposed for blood gas analysis. We analyzed pH, arterial partial pressure of oxygen (PaO_2_), arterial partial pressure of carbon dioxide (PaCO_2_), and arterial oxygen saturation (SaO_2_).

### 2.4 Trace fear conditioning test (FCT)

As previously described, the trace FCT has been found to be closely related to the memory associated with α5‐GABA_A_R (Crestani et al., 2002). Therefore, a trace FCT was performed 24 h after the mice were anesthetized. Briefly, mice were placed in a chamber and allowed to explore freely for 3 min to acclimatize to the environment. The mice were then subjected to four repetitions of tone–shock pairings (tone 3000 Hz, level 3, 30 s; foot shock 0.8 mA, 2 s). There was a 3 s interval between the tone and the shock. The interval between each cycle was 1 min. After the last shock, the mice were allowed to remain in the box for 1 min before being returned to their original cage. Twenty‐four hours after the training, the mice were returned to the same chamber for 6 min without any tone or shock. After another 2 h, the mice were placed in a new box with a different odor and environment and the same sound stimulus and interval settings as in the previous training session but without the shock stimuli. The freezing time of the mice was recorded separately.

### 2.5 Morris water maze (MWM)

In addition to the trace FCT, the MWM test was also performed as previously described (Vorhees & Williams, 2006; Wang, Meng, et al., 2018). The MWM is a very important technique for testing the learning and memory abilities of animals and has been found to be hippocampus dependent. Briefly, a circular pool with a diameter of 1.2 m was filled with water, and the water temperature was maintained between 22 and 24°C. The pool was marked with four entry points, N, S, E, and W, according to the spatial location, and divided into four quadrants. In quadrant II, a direct 10 cm platform was placed, and the platform height was 1 cm below the water surface. Mice were trained for 4 to 5 days (also called the navigation stage). During the navigation stage, the mice were placed in the pool at four different entry points each day for free exploration. If the mouse found the hidden platform within 60 s, that time was recorded as the escape latency of the mouse. If the mouse failed to find the platform within 60 s, it was guided to the platform and allowed to stay there for 30 s to remember the location of the platform. Twenty‐four hours after the last training, the platform was removed, and the mice were placed in the water for 60 s to explore freely. Escape latency, the time spent in quadrant II (target quadrant), and swimming speed were recorded and analyzed.

### 2.6 Drug treatment

L‐655708 (MedChemExpress, NJ, USA) was administered intraperitoneally as an inhibitor of α5‐GABA_A_R at a dose of 0.5 mg/kg 30 min before the onset of each behavioral test (Zuo et al., 2021). To inhibit ROCK2, fasudil hydrochloride (MedChemExpress, NJ, USA) was administered intraperitoneally at a dose of 20 mg/kg/day for 7 days before anesthesia (Zhu et al., 2022).

### 2.7 Stereotaxic injection

Mice were anesthetized with intraperitoneal pentobarbital sodium and placed on a brain stereotaxic instrument (Chongshi, China). The skin was cut to expose the skull, and the meninges were carefully dissected. The hippocampus was located (anterior‐posterior [AP], −2.00 mm; medial‐lateral [ML], ±1.80 mm). The microsyringe was slowly inserted at a depth of 1.80 mm from the dura mater for a 5 min stay, after which the device was returned to a depth of 1.60 mm. A total of 2 μL (1 μL per site) of AAV‐*rock2* or AAV‐control (Genechem, Shanghai, China) was injected into the bilateral hippocampus at 0.2 μL/min using a microinjection pump. The microsyringe was slowly returned to a depth of 1.40 mm for 5 min after a 5 min stay at the end of the injection, after which the microsyringe was withdrawn. The skull gap was closed with bone wax, and the skin was sutured following injection. Mice were returned to warm cages and allowed to acclimate for 4 weeks before subsequent experiments. Western blotting and immunofluorescence were used to evaluate the efficiency of the transfection.

### 2.8 Western blotting

Total proteins and membrane proteins were extracted from the mouse hippocampus for Western blot analysis. A Whole‐Cell Lysis Assay (Keygen Biotech, Jiangsu, China) and Membrane and Cytosol Protein Extraction Kit (Keygen Biotech, Jiangsu, China) were used to extract total proteins and membrane proteins from fresh hippocampal tissues, respectively, according to the manufacturer's instructions. After the proteins were extracted, SDS loading buffer was added to the protein samples, and the mixtures were boiled for 7 min. The protein samples were separated via SDS–PAGE and transferred to PVDF membranes. QuickBlock™ Blocking Buffer for Western Blotting (Beyotime Biotechnology, Shanghai, China) was used to block the PVDF membrane for 30 min at room temperature after the transfer. The corresponding primary antibody was diluted, added to the PVDF membrane, and incubated overnight at 4°C. After 14 h, the antibody was removed, the membrane was washed three times with TBST, and the membrane was incubated with the secondary antibody for 1 h at room temperature. After washing again with TBST, the results were analyzed using Image Lab software (Bio‐Rad, Hercules, CA, USA). The following antibodies were used: anti‐gephyrin (Abcam, ab177154, 1:1000), anti‐radixin (Abcam, ab52495, 1:1000), anti‐actin [EPR16769] (Abcam, ab179467, 1:5000), anti‐GABA receptor alpha 5 (Abcam, 259880, 1:1000), anti‐ROCK2 (middle) polyclonal (Proteintech, 21645‐1‐AP, 1:2000), goat anti‐rabbit IgG H&L (HRP) (Abcam, ab205718, 1:10,000), and anti‐sodium potassium ATPase (Abcam, ab76020, 1: 100,000).

### 2.9 Immunofluorescence

The mice were anesthetized and perfused transcardially with 0.9% saline and 4% paraformaldehyde. The brains of the mice were completely removed and immersed in 4% paraformaldehyde. After paraffin embedding, each mouse brain was cut into 5 μm sections. The slices were dewaxed and rehydrated with xylene and graded ethanol, rinsed with distilled water for 5 min, and then rinsed three times with PBS. After antigen retrieval using citrate, the sections were treated with 0.3% Triton X‐100 in a 37°C incubator for 30 min. Subsequently, the sections were blocked using a blocking solution for 30 min at room temperature and incubated in a primary antibody mixture at 4°C overnight. The sections were rinsed three times and incubated with the secondary antibody mixture for 2 h at room temperature. The following antibodies were used: anti‐phospho‐ezrin (Thr567)/radixin (Thr564)/moesin (Thr558) (48G2) rabbit mAb (Cell Signaling Technology, 3726, 1:200), anti‐GABA A receptor alpha 5 (Abcam, ab242001, 1:200), goat anti‐rabbit IgG H&L (Alexa Fluor 555) (Abcam, ab150078, 1:500), and anti‐mouse IgG H&L (Alexa Fluor 647) (Abcam, ab150115, 1:500). After the slides were sealed with DAPI‐containing anti‐fluorescence quenching solution (Servicebio, G1407) and covered with coverslips, the slices were placed under an underlaser‐focusing microscope (Zeiss LSM880, Germany) for imaging observation.

### 2.10 Statistical analysis

GraphPad Prism 8.3 software (GraphPad Prism Co., San Diego, CA, USA) was used to analyze the data. Comparisons between two groups were analyzed by two‐tailed Student's *t*‐tests. One‐way analysis of variance (ANOVA) was used to compare more than two groups of data, followed by Tukey's multiple comparisons test for inter‐group comparisons. Repeated measures of two‐way ANOVA followed by Tukey's multiple comparisons test were used to analyze the escape latency data from the MWM test, with days as the repeated factor. The specific details of the statistical analysis methods used for the experiments are provided in the figure legends. All the data are presented as the mean ± SEM. *p* < 0.05 was considered to indicate statistical significance.

## 3 RESULTS

### 3.1 Sevoflurane exposure impairs hippocampus‐dependent cognitive function in aged mice

Blood gas analysis was used to assess respiratory function in the mice. There were no significant differences in pH, PaO_2_, PaCO_2_, or SaO_2_ between the sevoflurane group and the control group for either young or aged mice (Tables 1 and 2) (*p* > 0.05).

**TABLE 1:** Blood gas analysis—Young mice (mean ± SEM, *n* = 7).

Group	CON‐young	SEV‐young	*p* Value
pH	7.33 ± 0.04	7.30 ± 0.03	0.55
PaCO_2_ (mmHg)	41.3 ± 4.14	44.0 ± 3.88	0.64
PaO_2_ (mmHg)	123.4 ± 8.09	122.4 ± 7.35	0.93
SaO_2_ (%)	95.7 ± 0.73	96.4 ± 0.87	0.55

**TABLE 2:** Blood gas analysis—Aged mice (mean ± SEM, *n* = 5).

Group	CON	SEV	*p* Value
pH	7.49 ± 0.07	7.40 ± 0.04	0.27
PaCO_2_ (mmHg)	30.7 ± 5.21	37.0 ± 2.81	0.31
PaO_2_ (mmHg)	105.1 ± 10.06	98.5 ± 6.72	0.60
SaO_2_ (%)	95.9 ± 1.82	93.0 ± 1.48	0.25

**FIGURE 1:**
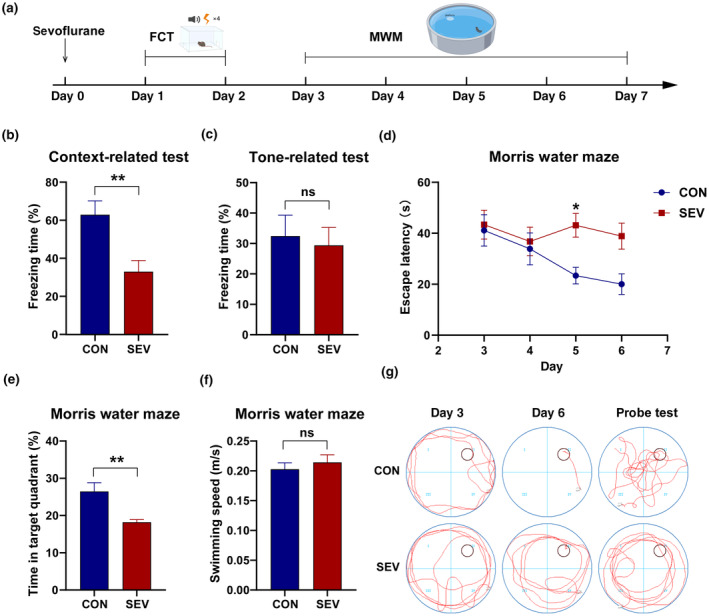
Sevoflurane impairs the cognitive function of aged mice. (a) Timeline of the experiment. (b, c) Freezing time of aged mice during the context‐related test and tone‐related test (*n* = 7). (d) Escape latency in the training phase (*n* = 7). (e, f) Time spent in the target quadrant and the swimming speed of aged mice in the probe test (*n* = 7). (g) Representative traces in the Morris water maze. The escape latency data were analyzed by repeated measures of two‐way ANOVA followed by Tukey's multiple comparisons test. All other data were analyzed using an unpaired Student's *t*‐test. The data are presented as the mean ± SEM. *p < 0.05, **p < 0.01. CON, control group; SEV, sevoflurane group.

To assess the effects of 4% sevoflurane exposure on the cognitive function of aged mice, we performed the FCT and MWM. Twenty‐four hours after exposure to sevoflurane, the mice were trained on FCT. The context‐related test and the tone‐related test were performed at 24 h and 26 h after the end of the training. In the tone‐related test, the freezing time of sevoflurane‐exposed mice was comparable to that of control mice (Figure 1c) (*p* > 0.05). However, in the context‐related test, there was a significant decrease in freezing time in aged mice after sevoflurane anesthesia (Figure 1b) (*p* < 0.01). Since memory analyzed in the tone‐related test is jointly regulated by the amygdala and hippocampus, whereas memory analyzed in the contextual test is mainly regulated by the hippocampus, the FCT results suggest that the cognitive impairment induced by sevoflurane exposure is mainly associated with the hippocampus.

To further verify the effect of sevoflurane on hippocampus‐related cognitive function in aged mice, we performed the MWM test. On day 5, the escape latency of the sevoflurane group was significantly longer than that of the control group (Figure 1d) (*p* < 0.05). In the probe test, there was no significant difference in the swimming speed of the mice in the different groups (Figure 1f), but the sevoflurane‐treated aged mice spent significantly less time in the target quadrant than did the control group (Figure 1e) (*p* < 0.01), demonstrating that sevoflurane exposure impaired the memory of the platform location in aged mice.

These results suggest that exposure to 4% sevoflurane severely affects hippocampus‐related cognitive function in aged mice.

### 3.2 Sevoflurane induces the overexpression of α5‐GABA_A_R in aged mice

**FIGURE 2:**
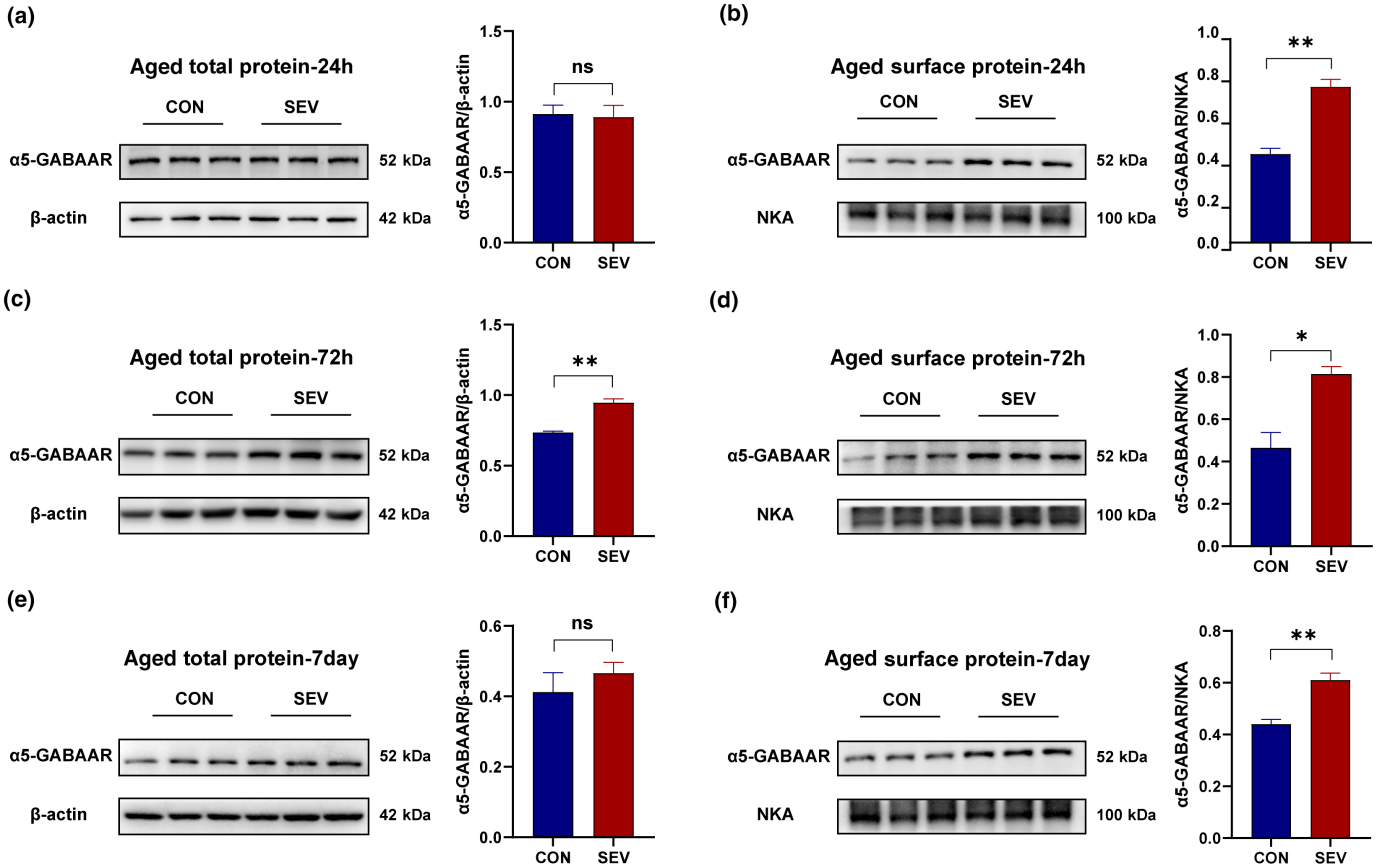
Sevoflurane induces the overexpression of α5‐GABA_A_R on the cell membrane in aged mice. (a, c, e) The expression of α5‐GABA_A_R in hippocampal total protein samples from aged mice (*n* = 3 per group). β‐Actin was used as the loading control for total protein. (b, d, f) The expression of α5‐GABA_A_R in hippocampal membrane protein samples from aged mice (*n* = 3 per group). NKA was used as the loading control for the membrane protein samples. All the data were analyzed using an unpaired Student's *t*‐test. *p < 0.05, **p < 0.01. NKA, sodium potassium ATPase.

To examine the changes in α5‐GABA_A_R expression in hippocampal tissue after sevoflurane inhalation, we extracted total and membrane proteins. By comparing the levels of β‐actin to those of the membrane proteins and total proteins, we determined the reliability of the membrane protein extraction method (Figure S1) (*p* < 0.0001). We extracted the relevant proteins at 24 h, 72 h, and 7 days after sevoflurane exposure. As shown in Figure 2, the expression of α5‐GABA_A_R in the total protein samples was significantly increased in aged mice at 72 h after sevoflurane treatment (Figure 2c) (*p* < 0.01), but by day 7, α5‐GABA_A_R expression in the total protein samples had returned to a normal level (Figure 2e). However, in the membrane, an increase in the α5‐GABA_A_R concentration occurred 24 h after anesthesia, with this increase persisting until day 7 (Figure 2b,d,f) (24 h, *p* < 0.01; 72 h, *p* < 0.05; 7 days, *p* < 0.01); this is consistent with the behavioral manifestations of cognitive impairment over time.

### 3.3 Sevoflurane has no significant effect on α5‐GABA_A_R expression in young mice

To investigate the effect of sevoflurane exposure under the same conditions on young mice, we also performed the FCT and MWM test. The behavioral results (Figure S2) showed that sevoflurane treatment under the same conditions did not cause cognitive dysfunction in young mice; therefore, we also analyzed the expression of α5‐GABA_A_R in young mice. As shown in Figure S3, from 24 h until the 7th day after sevoflurane exposure, there was no significant difference in the expression of α5‐GABA_A_R in young mice compared to that in the control group in either the total protein or membrane protein fraction. These results matched the behavioral findings.

These results suggested that α5‐GABA_A_R overexpression on the cell membrane might be a key target for treating sevoflurane‐induced cognitive impairment in aged mice.

### 3.4 L‐655708 reverses sevoflurane‐induced cognitive dysfunction in aged mice

**FIGURE 3:**
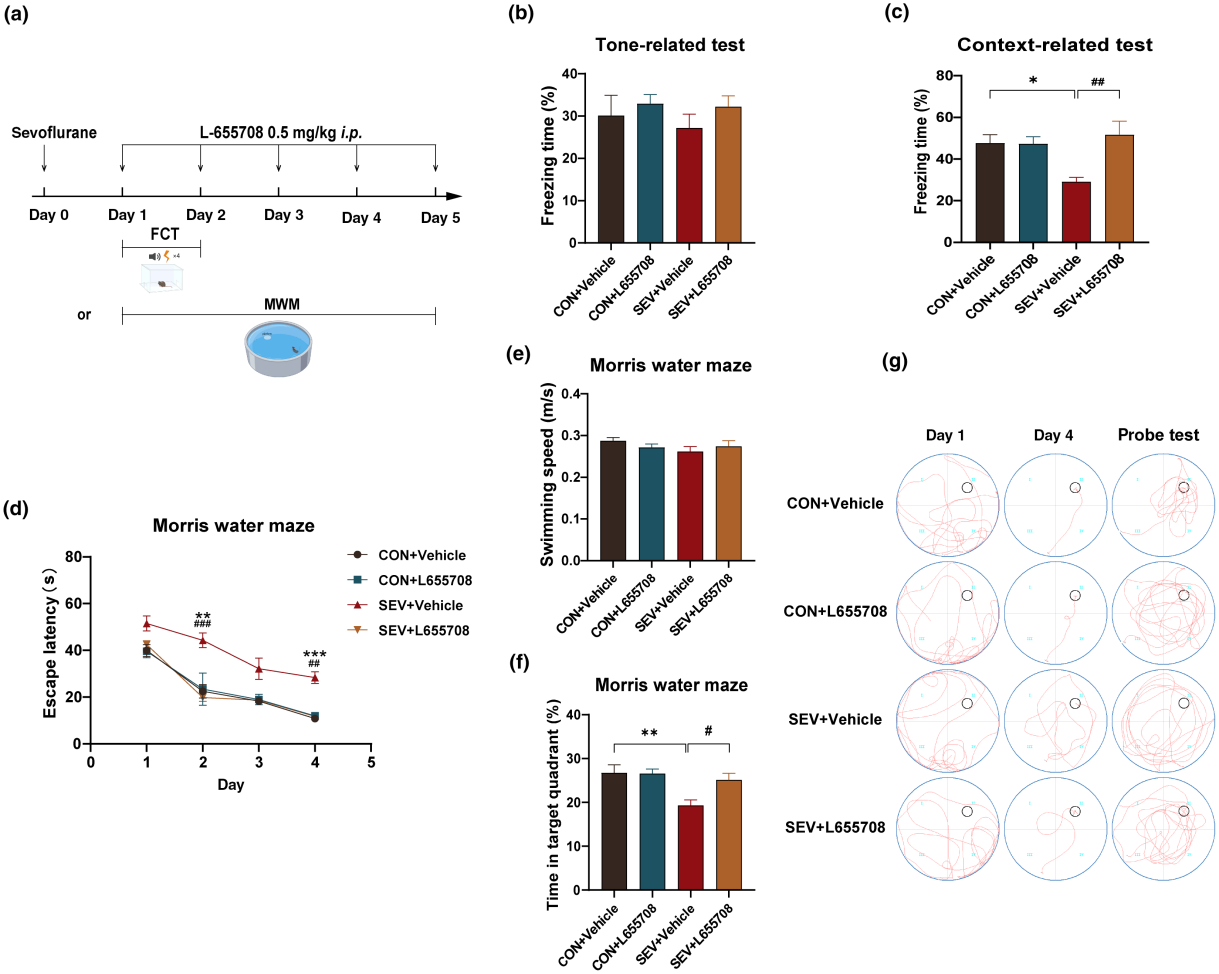
Inhibiting the function of α5‐GABA_A_R reverses sevoflurane‐induced cognitive dysfunction. (a) The process of the experience. The FCT or MWM test was performed 24 h after anesthesia. L‐655708 or vehicle was used 0.5 h before every behavioral test. The mice that underwent the FCT and MWM tests were different (*n* = 7 per group). (b, c) Freezing time during the context‐related test and tone‐related test (*n* = 7). (d) Escape latency in the training phase (*n* = 7 per group). (e, f) Swimming speed and time spent in the target quadrant during the probe test (*n* = 7 per group). (g) Representative traces in the Morris water maze. The escape latency (d) was analyzed with two‐way ANOVA followed by Tukey's multiple comparisons test. All other data were analyzed with one‐way ANOVA followed by Tukey's multiple comparisons test. The data are presented as the mean ± SEM. *p < 0.05, **p < 0.01, ***p < 0.001; ^#^ *p* < 0.05, ^##^ *p* < 0.01, ^###^ *p* < 0.001. FCT, fear conditioning test; MWM, Morris water maze.

Through molecular experiments, we found that the amount of α5‐GABA_A_R on the membrane was significantly greater in sevoflurane‐treated aged mice than in control mice. To further verify the role of this molecule in the development of cognitive impairment, we used L‐655708, an inhibitor of α5‐GABA_A_R that has been shown to specifically inhibit the function of α5‐GABA_A_R in numerous studies, to inhibit the function of α5‐GABA_A_R. In the FCT, the freezing time of aged mice treated with sevoflurane and L‐655708 (SEV+L‐655708) was significantly longer than that of aged mice treated with sevoflurane alone (SEV+Vehicle) (*p* < 0.01), demonstrating that inhibiting the function of α5‐GABA_A_R improved the cognitive function of aged mice (Figure 3b,c). Moreover, we conducted the MWM test for verification. During training, aged mice exhibited a longer escape latency after sevoflurane exposure alone than the control group (CON+Vehicle) (day 2, *p* < 0.01; day 4, *p* < 0.001), but this change was reversed by L‐655708 (day 2, *p* < 0.001; day 4, *p* < 0.01) (Figure 3d). During the probe test phase, the aged mice in the SEV+Vehicle group had a significantly shorter stay in the target quadrant than did those in the CON+Vehicle group (*p* < 0.01), while the memory for the platform location was restored in the anesthetized mice treated with L‐655708 (*p* < 0.05) (Figure 3f).

### 3.5 Sevoflurane increases the expression of extrasynaptic α5‐GABA_A_R

**FIGURE 4:**
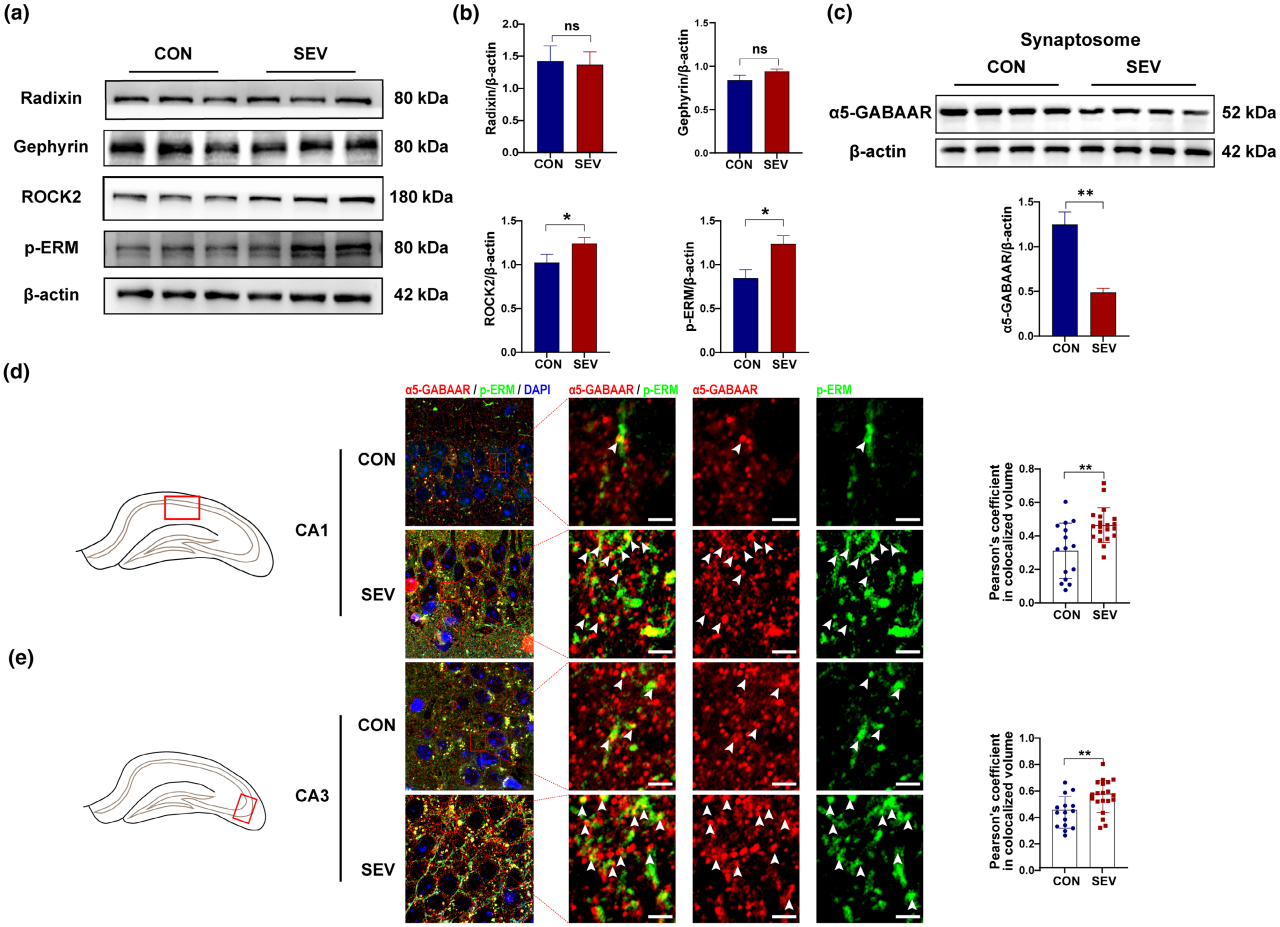
Sevoflurane increases the distribution of extrasynaptic α5‐GABA_A_R by affecting the phosphorylation of radixin. (a) Representative images of Western blots. (b) The level of phosphorylation of radixin and the expression of ROCK2, gephyrin, and radixin (*n* = 3 per group). (c) Representative bands in the synaptosome. The expression of synaptic α5‐GABA_A_R decreased significantly after exposure to sevoflurane (*n* = 4 per group). (d, e) Representative immunofluorescence images showing the colocalization of α5‐GABA_A_R (red) and p‐ERM (green) in the CA1 region and CA3 region, respectively. Arrows: the regions in which p‐ERM and α5‐GABA_A_R were colocalized. Scale bars, 2 μm. All data were analyzed using an unpaired Student's *t*‐test. The data are presented as the mean ± SEM. *p < 0.05, **p < 0.01. p‐ERM, phosphorylated form of radixin.

As one of the most important receptors at inhibitory synapses, α5‐GABA_A_R is found at both extrasynaptic and synaptic sites but most predominantly at extrasynaptic sites. To determine whether the increase in α5‐GABA_A_R membrane expression induced by sevoflurane preferentially occurred at synaptic or extrasynaptic sites, synaptosomes were prepared. Sevoflurane treatment resulted in the downregulation of α5‐GABA_A_R expression at synaptic sites (Figure 4c) (*p* < 0.01). Moreover, immunostaining for p‐ERM, which indicates the phosphorylation of radixin and α5‐GABA_A_R, was performed to observe the expression of extrasynaptic receptors. We found that the degree to which p‐ERM colocalized with α5‐GABA_A_R was significantly greater in the sevoflurane group than in the control group (Figure 4d,e) (CA1, *p* < 0.01; CA3, *p* < 0.01). Next, we determined the expression of gephyrin and radixin, two anchors of α5‐GABA_A_R at synaptic and extrasynaptic sites, respectively. However, there was no significant difference in the expression of these two proteins after sevoflurane treatment (Figure 4a). Considering that radixin, an extrasynaptic anchor protein, must be phosphorylated to exert its function, we examined the phosphorylation level of this protein. The results suggested that sevoflurane exposure increased radixin phosphorylation (Figure 4a) (*p* < 0.05). These results demonstrated that sevoflurane anesthesia increased the phosphorylation of the extrasynaptic anchoring protein radixin and that the α5‐GABA_A_R was more strongly anchored to extrasynaptic sites.

### 3.6 The RhoA/ROCK2 signaling pathway is involved in the membrane expression of the α5‐GABA_A_R

**FIGURE 5:**
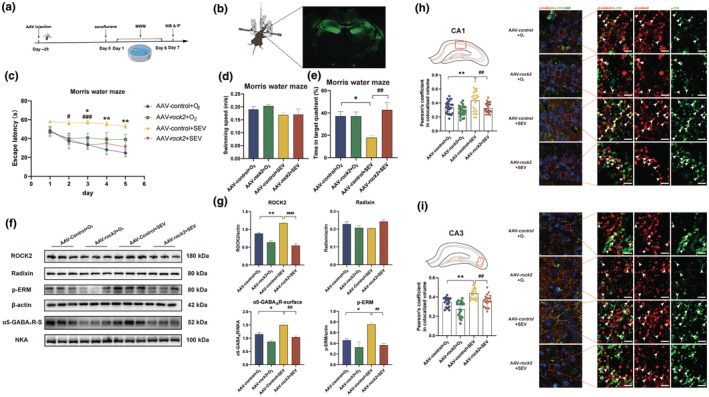
Cognitive impairment and the abnormal expression of extrasynaptic α5‐GABA_A_R are influenced by the RhoA/ROCK2 signaling pathway. (a) Timeline of the experiment. (b) Stereotaxic injection. The fluorescence image shows the location of the AAV, confirming that the injection site was correct. (c–e) Morris water maze. (c) Escape latency in the training stage (*n* = 6 per group). (d, e) Swimming speed and time spent in the target quadrant during the probe test (*n* = 6 per group). (f) Western blot images of ROCK2, radixin, and p‐ERM bands in total protein samples and α5‐GABA_A_R bands in membrane protein samples (*n* = 3). β‐Actin and NKA were used as total protein and membrane protein loading controls, respectively. (g) The corresponding densitometry analysis of ROCK2, radixin, and p‐ERM for total protein samples and α5‐GABA_A_R for membrane protein samples (*n* = 3). (h, i) The colocalization of p‐ERM (green) and α5‐GABA_A_R (red). Arrow: The region in which p‐ERM and α5‐GABA_A_R were colocalized (yellow). The degree of colocalization in CA1 (h) and CA3 (i) is shown by Pearson's coefficient. The escape latency (c) was analyzed with two‐way ANOVA followed by Tukey's multiple comparisons test. All other data were analyzed with one‐way ANOVA followed by Tukey's multiple comparisons test. The data are presented as the mean ± SEM. *p < 0.05, **p < 0.01; ^#^ *p* < 0.05, ^##^ *p* < 0.01, ^###^ *p* < 0.001. p‐ERM, phosphorylated form of radixin.

The phosphorylation of radixin is regulated mainly by the RhoA/ROCK2 signaling pathway. To investigate the changes in the RhoA/ROCK2 signaling pathway after sevoflurane treatment, we first observed the expression of ROCK2. Figure 4a shows a significant increase in ROCK2 protein expression in aged mice treated with sevoflurane compared with that in the control group (*p* < 0.05). Therefore, the role of the RhoA/ROCK2 signaling pathway in cognitive function was demonstrated. We constructed an adeno‐associated virus (AAV) that specifically interfered with *rock2* gene expression (Figure 5a,b). Four weeks after the targeted injection of the virus into the bilateral hippocampus, the mice were exposed to sevoflurane. The MWM test results showed that the escape latency of the AAV‐control+SEV group was significantly longer than that of the control group during the training period (day3, *p* < 0.05; day 4, *p* < 0.01; day 5, *p* < 0.01) and the latency was significantly shorter after specific knockdown of *rock2* (day 2, *p* < 0.05; day 3, *p* < 0.001) (Figure 5c). Moreover, in the probe test, sevoflurane significantly reduced the residence time in the target quadrant at comparable swimming speeds (*p* < 0.05), while sevoflurane did not cause such a change in anesthetized mice injected with AAV‐*rock2* (Figure 5d,e). These results suggested that the RhoA/ROCK2 signaling pathway was involved in sevoflurane‐induced cognitive impairment.

To investigate whether the RhoA/ROCK2 signaling pathway plays a role in affecting the phosphorylation of radixin, Western blotting was used to determine the phosphorylation level of radixin and the expression of α5‐GABA_A_R. As expected, sevoflurane‐induced aberrant phosphorylation of radixin was blocked by interference with the *rock2* gene (*p* < 0.01). Moreover, the plasma membrane α5‐GABA_A_R concentration decreased significantly (*p* < 0.01) (Figure 5f,g). Next, we used an immunofluorescence technique to observe the changes in the expression of extrasynaptic α5‐GABA_A_R. The immunofluorescence results suggested that the sevoflurane‐induced increase in the colocalization of α5‐GABA_A_R and p‐radixin in both the CA1 (*p* < 0.01) and CA3 (*p* < 0.01) (Figure 5h,i) regions of the hippocampus was prevented after the injection of AAV (CA1, *p* < 0.01; CA3, *p* < 0.01).

### 3.7 Fasudil hydrochloride reverses sevoflurane‐induced cognitive dysfunction in aged mice

**FIGURE 6:**
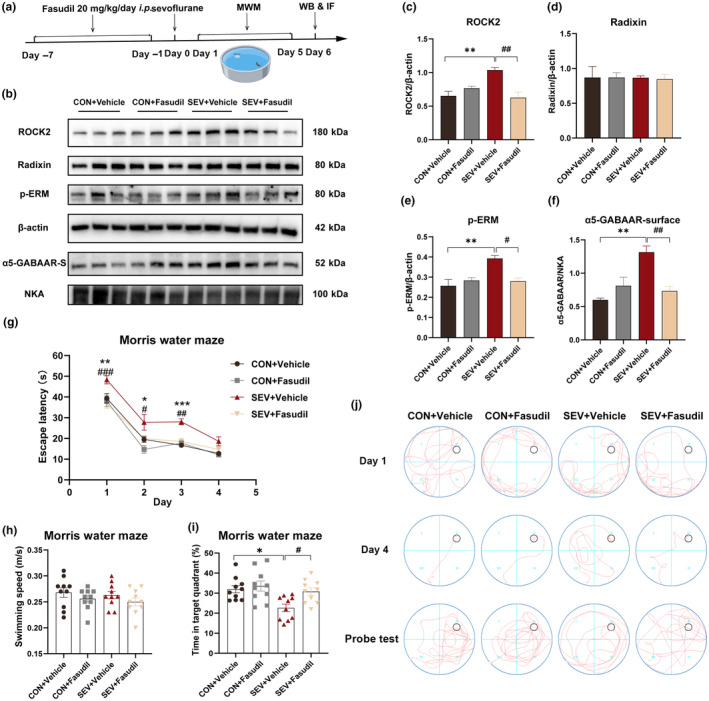
Fasudil hydrochloride inhibits the abnormal surface expression of α5‐GABA_A_R and improves cognitive function. (a) Timeline of experience. (b) Western blot images of ROCK2, radixin, and p‐ERM bands in total protein samples and α5‐GABA_A_R bands in membrane protein samples (*n* = 3 per group). β‐Actin and NKA were used as total protein and membrane protein loading controls, respectively. (c–f) The corresponding densitometry analysis of ROCK2, radixin, and p‐ERM in total protein samples and of α5‐GABA_A_R in surface protein samples (normalized to β‐actin or NKA) (*n* = 3). (g) Escape latency in the training stage (*n* = 10). (h, i) Swimming speed and time spent in the target quadrant during the probe test (*n* = 10). (j) Representative traces of the four groups in the Morris water maze. The escape latency (g) was analyzed with two‐way ANOVA followed by Tukey's multiple comparisons test. All other data were analyzed with one‐way ANOVA followed by Tukey's multiple comparisons test. The data are presented as the mean ± SEM. *p < 0.05, **p < 0.01, ***p < 0.001; ^#^ *p* < 0.05, ^##^ *p* < 0.01, ^###^ *p* < 0.001. IF, immunofluorescence; MWM, Morris water maze; p‐ERM, phosphorylated form of radixin; WB, Western blot.

Fasudil hydrochloride, a ROCK2 inhibitor, has been used clinically to improve cerebral vasospasm after subarachnoid hemorrhage. To test whether fasudil hydrochloride could improve sevoflurane‐induced cognitive impairment, fasudil hydrochloride was injected intraperitoneally into mice at a dose of 20 mg/kg 1 week prior to sevoflurane exposure (Figure 6a). During the training process, mice treated with both fasudil and sevoflurane (SEV+fasudil) had significantly shorter escape latencies than did mice anesthetized with sevoflurane alone (SEV+Vehicle) (Figure 6g) (day 1, *p* < 0.01; day 2, *p* < 0.05; day 3, *p* < 0.001). Moreover, fasudil hydrochloride significantly improved the residence time of the mice in the target quadrant in the probe test without affecting their swimming speed (Figure 6h,i) (*p* < 0.05). Western blot analysis revealed that, compared with that in the SEV+Vehicle group, the level of phosphorylated radixin in the SEV+fasudil group was significantly lower (*p* < 0.05). Moreover, the sevoflurane‐induced increase in the α5‐GABA_A_R concentration on the plasma membrane (*p* < 0.01) was also prevented by fasudil hydrochloride (*p* < 0.01) (Figure 6b–f). These results indicate that fasudil hydrochloride could serve as a clinical inhibitor of ROCK2 and ameliorate the cognitive impairment induced by sevoflurane in elderly mice.

## 4 DISCUSSION

In the present study, we found that aged mice exposed to 4% sevoflurane exhibited a decrease in spatial cognitive function accompanied by an increase in α5‐GABA_A_R expression on the cell membrane. However, young mice treated with the same dose of sevoflurane showed no changes in cognition or in α5‐GABA_A_R expression. With the use of L‐655708, an inhibitor of the α5‐GABA_A_R, we found that cognitive function was improved in aged mice exposed to sevoflurane. More importantly, in this study, we clearly demonstrated for the first time that extrasynaptic α5‐GABA_A_R shows increased expression following sevoflurane exposure. Additionally, intervention in the RhoA/ROCK2 signaling pathway via pharmacological approaches and genetic modification attenuates the phosphorylation of radixin, thereby mitigating cognitive impairments induced by sevoflurane.

GABAergic synapses are the most important inhibitory synapses and play a key role in maintaining the balance between excitation and inhibition in the central nervous system. During the action of benzodiazepines, GABA type A receptors, which contain different types of subunits, exert different effects. Receptors containing the α1 subunit are related to sedation and hypnosis, and those containing α2 and α3 have anxiolytic effects (Mohamad & Has, 2019). However, α5‐GABA_A_R has been found to be associated with cognitive function. In addition, in several other neurological disorders, α5‐GABA_A_R has been found to affect cognitive function in laboratory animals (Clarkson et al., 2010; Martínez‐Cué et al., 2013; Xu & Wong, 2018). Therefore, increasing amounts of attention have been focused on α5‐GABA_A_R. In this study, we found that an abnormal increase in α5‐GABA_A_R expression on the cell membrane was accompanied by a decrease in cognitive function in aged mice after sevoflurane inhalation. Moreover, inhibiting the function of α5‐GABA_A_R with L‐655708, an inhibitor of α5‐GABA_A_R, significantly improved cognitive function in aged mice, which is consistent with the findings of previous studies (Zhang et al., 2020; Zuo et al., 2021; Zurek et al., 2014). These findings revealed that the aberrant expression of α5‐GABA_A_R on the cell membrane plays a pivotal role in the cognitive deficits induced by sevoflurane.

At the subcellular level, GABA_A_Rs can be divided into synaptic receptors and extrasynaptic receptors that mediate phasic inhibition and tonic inhibition, respectively (Jacob, 2019; Prévot & Sibille, 2021). Research has revealed that α5‐GABA_A_R is present at both synaptic and extrasynaptic sites, with predominant distribution at extrasynaptic sites. In addition, gephyrin and radixin serve as anchor proteins for α5‐GABA_A_R, facilitating the localization of the receptors to synaptic and extrasynaptic sites, respectively (Caraiscos et al., 2004; Hannan et al., 2020; Martin et al., 2010). Therefore, in the present study, we investigated the expression of these two anchor proteins to determine whether the sevoflurane‐induced increase in α5‐GABA_A_R expression occurred predominantly at synaptic or extrasynaptic sites. However, our results did not reveal any changes.

Radixin, the anchor protein for extrasynaptic α5‐GABA_A_R, needs to be phosphorylated to carry out its anchoring role. The phosphorylation of radixin is orchestrated through the RhoA/ROCK2 signaling pathway (Hausrat et al., 2015; Loebrich et al., 2006). In this study, we found that the phosphorylation of radixin was significantly increased, and this change was accompanied by activation of the RhoA/ROCK2 signaling pathway. Moreover, the colocalization of α5‐GABA_A_R and p‐ERM was also significantly increased. These results suggest that the increase in the number of receptors on the cell membrane mainly occurred at extrasynaptic sites, which is in line with the tonic current enhancement in the hippocampus after anesthesia observed by Zurek et al. (2014) and offers a theoretical explanation for the molecular underpinnings of these electrophysiological modifications.

The RhoA/ROCK2 signaling cascade has emerged as a pivotal player in the pathogenesis of a spectrum of diseases. In contemporary research, the involvement of this cascade in Alzheimer's disease has been progressively corroborated, underscoring its integral role in the pathology of cognitive dysfunction (Zhu et al., 2022). Furthermore, it has been revealed that sevoflurane can activate this signaling pathway via different mechanisms. Yu et al. (2003) reported that sevoflurane can inhibit the transfer of RhoA/ROCK2 to the cell membrane in a dose‐dependent manner, thereby inhibiting aortic smooth muscle contraction. Other studies revealed that sevoflurane impairs cognitive function via the proBDNF‐p75NTR‐RhoA pathway or SIRT1/RhoA pathway (Dong et al., 2020; Zhou et al., 2022). In this investigation, we focused on the impact of sevoflurane on the subcellular localization of α5‐GABA_A_R through the RhoA/ROCK2 signaling pathway. Consequently, we refrained from an exhaustive exploration of the methodologies by which sevoflurane influences the RhoA/ROCK2 signaling cascade. Hence, additional studies are warranted to elucidate the influence of sevoflurane on the RhoA/ROCK2 signaling pathway within our model framework.

Fasudil hydrochloride is a vasodilator used for the prevention and treatment of cerebral vasospasm. Additionally, fasudil is an inhibitor of ROCK2. In recent years, fasudil hydrochloride has been widely used in animal experiments, where its inhibitory effect on ROCK2 and its safety in animals have been effectively validated (Xu et al., 2021; Yan et al., 2015). Using prophylactic administration of fasudil hydrochloride, we found that cognitive impairment in aged mice following sevoflurane treatment was reversed. Zhu et al. (2022) discovered that fasudil alleviated acrolein‐induced learning and memory impairment, which was to some extent consistent with our findings. The revelation of the ability of fasudil, a pharmaceutical already available in the marketplace, to enhance cognitive function is a noteworthy clinical finding of this investigation. This study potentially provides a novel strategy for the prevention and management of PND.

PND refers to central nervous system complications after anesthesia surgery and mainly manifests as cognitive decline. Although PND has been studied for many years, the specific pathogenesis of PND has not been determined. A large‐scale retrospective clinical study revealed that the incidence of dementia in elderly patients receiving inhalation anesthesia is significantly greater than that in elderly patients receiving total intravenous anesthesia or regional block anesthesia (Sun et al., 2023), which suggests that inhalation anesthetics has detrimental effects on cognition. Sevoflurane, a common inhalation anesthetic, has also been found to increase the incidence of PND in clinical trials. Due to ethical limitations, it is not feasible to conduct clinical research solely using sevoflurane; thus, the study of the effects of sevoflurane on cognition in animals is highly important. In this study, we found that aged mice exhibited significant impairment of spatial learning and memory after anesthesia, which demonstrated the significant role of sevoflurane in cognition. Although the cognitive impairment caused by sevoflurane differs from PND observed in clinical settings, clarifying the role of sevoflurane in cognitive impairment can aid in the exploration of the pathogenesis of PND. In addition, we used an inhaled sevoflurane concentration of 4% in our investigation. Numerous studies have confirmed that the minimum alveolar concentration (MAC) of sevoflurane is approximately 3.3% in mice (Koyama et al., 2009; Liao et al., 2006; Petrenko et al., 2007). Therefore, a concentration of 4% is close to 1.3 MAC for mice, which corresponds to the concentration of sevoflurane commonly used for maintenance of anesthesia in clinical practice and better simulates the effect of sevoflurane in the clinic. Moreover, by removing the influence of surgical variables, we provide theoretical underpinnings evidence for anesthesiologists aiming to employ sevoflurane more safely.

This study has several limitations. First, we focused on the extrasynaptic α5‐GABA_A_R and related pathways. The distribution of α5‐GABA_A_R at synaptic sites and the related regulatory mechanisms have not been studied in detail. The expression and distribution of GABA type A receptors are extremely complex processes, and the regulation of α5‐GABA_A_R at synaptic sites and its extrasynaptic distribution are also considered mutually independent processes. Since α5‐GABA_A_R is mainly distributed in the extrasynaptic region and because gephyrin, a synaptic anchor, was not differentially expressed, we focused on the pathway involved in regulating the distribution of extrasynaptic receptors. However, further research is needed to determine whether the synaptic position of α5‐GABA_A_R and whether the regulation of its distribution are involved in sevoflurane‐induced cognitive impairment. Second, the lack of electrophysiological data was also a very regrettable finding in this study. We focused on the understanding of the mice, which was why we considered that behavioral experiments were sufficient to replace electrophysiology to determine the role of α5‐GABA_A_R. Additionally, this study did not explore the specific mechanisms by which sevoflurane induces changes in the RhoA/ROCK2 signaling pathway, necessitating further investigation and experimental validation.

In conclusion, sevoflurane anesthesia alone can induce cognitive impairment in aged mice by upregulating the expression of α5‐GABA_A_R at extrasynaptic sites. Sevoflurane can hyperactivate the RhoA/ROCK2 signaling pathway in the hippocampus, which in turn increases the phosphorylation of radixin, the anchor protein of extrasynaptic α5‐GABA_A_R. Fasudil hydrochloride, an inhibitor of the RhoA/ROCK2 signaling pathway, inhibits overactivation of the RhoA/ROCK2 pathway, thereby improving cognitive function in aged mice. Therefore, our study provides a potential target for determining the pathogenesis of PND and exploring strategies for the prevention and treatment of PND.

## AUTHOR CONTRIBUTIONS

Yiqing Yin and Yongan Wang designed the experiments. Zhun Wang and Jinpeng Dong contributed to writing the manuscript. Zhun Wang, Jinpeng Dong, and Mengxue Zhang contributed to performing the experiments. Shengran Wang and Sixuan Wang contributed to the data analysis. Jiangnan Wu and Yuan Luo contributed to editing the images and revising the manuscript. All authors contributed to the article and approved the submitted version.

## FUNDING INFORMATION

This work was supported by the Tianjin Scientific Research Strat‐up Foundation for Talent Introduction (No. 2021‐1‐10) and the “14th Five‐Year Plan” Peak Discipline Support Plan of Tianjin Medical University Cancer Institute and Hospital (NO. 7‐2‐13). This work was also funded by the Tianjin Key Medical Discipline (Specialty) Construction Project (TJYXZDXK‐009A).

## CONFLICT OF INTEREST STATEMENT

There are no conflicts of interest to disclose.

## Supporting information


Figure S1.

Figure S2.

Figure S3.

## Data Availability

The data that support the findings of this study are available from the corresponding author upon reasonable request.
