# Supplementary material for: Sevoflurane‐induced overexpression of extrasynaptic α5‐GABAAR via the RhoA/ROCK2 pathway impairs cognitive function in aged mice
Source: Aging Cell. 2024 Jun 2;23(9):e14209. doi: 10.1111/acel.14209 (PMC11488297; doi:10.1111/acel.14209)
Supplement: Supplementary file 1 — Figure S1. [file ACEL-23-e14209-s002.zip › FigureS1Caption.docx]

Figure S1. The expression of β-actin in total protein and membrane surface protein. NKA was used as the loading control. All the data were analyzed using an unpaired Student's *t*-test. **** *p* < 0.0001. NKA, sodium potassium ATPase.
