# Supplementary figures and images for: Sevoflurane‐induced overexpression of extrasynaptic α5‐GABAAR via the RhoA/ROCK2 pathway impairs cognitive function in aged mice
Source: Aging Cell. 2024 Jun 2;23(9):e14209. doi: 10.1111/acel.14209 (PMC11488297; doi:10.1111/acel.14209)

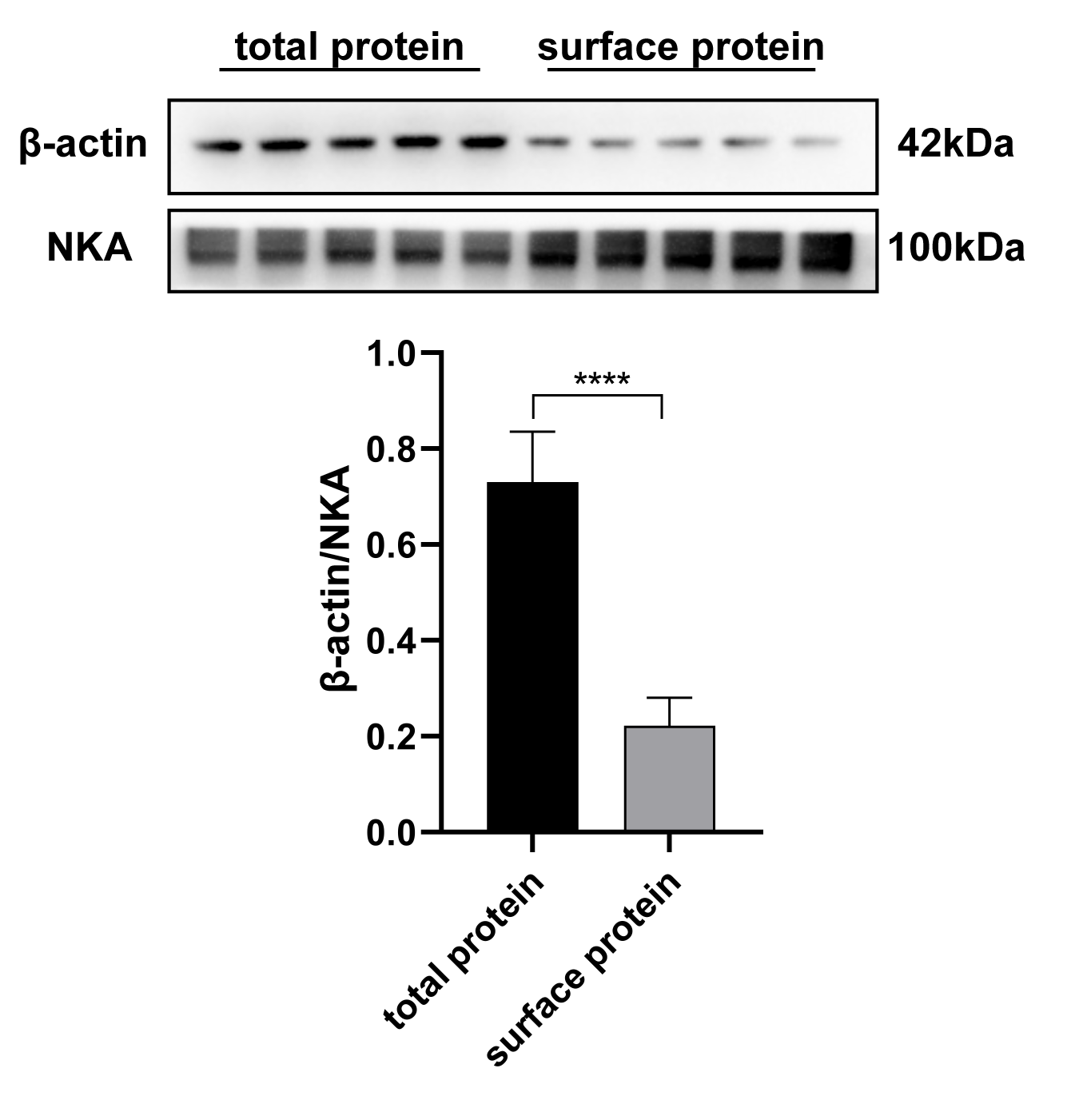

Supplement: Supplementary file 1 — Figure S1. [file ACEL-23-e14209-s002.zip › acel14209-sup-0001-FigureS1.tif]

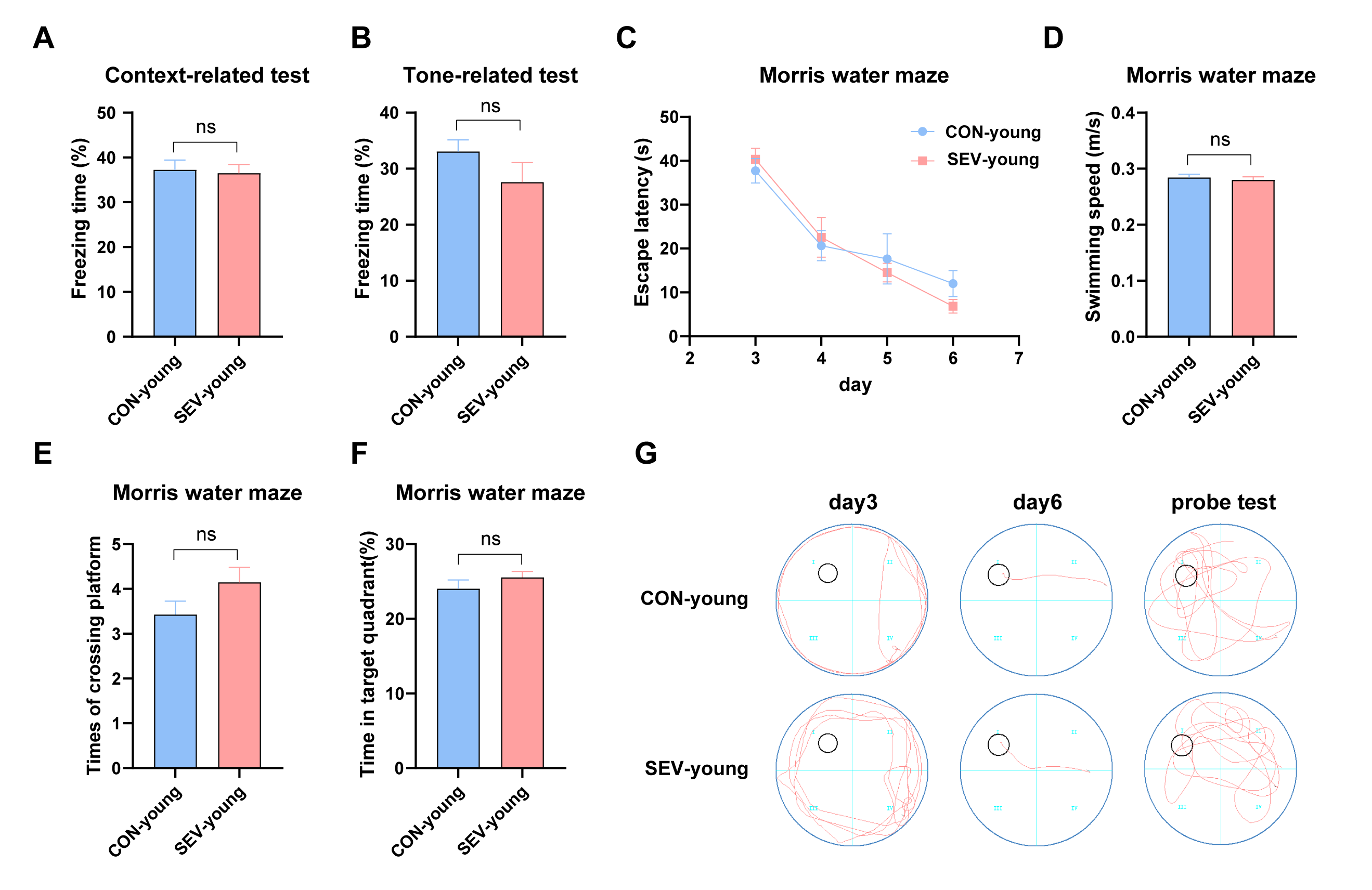

Supplement: Supplementary file 2 — Figure S2. [file ACEL-23-e14209-s003.zip › acel14209-sup-0002-FigureS2.tif]

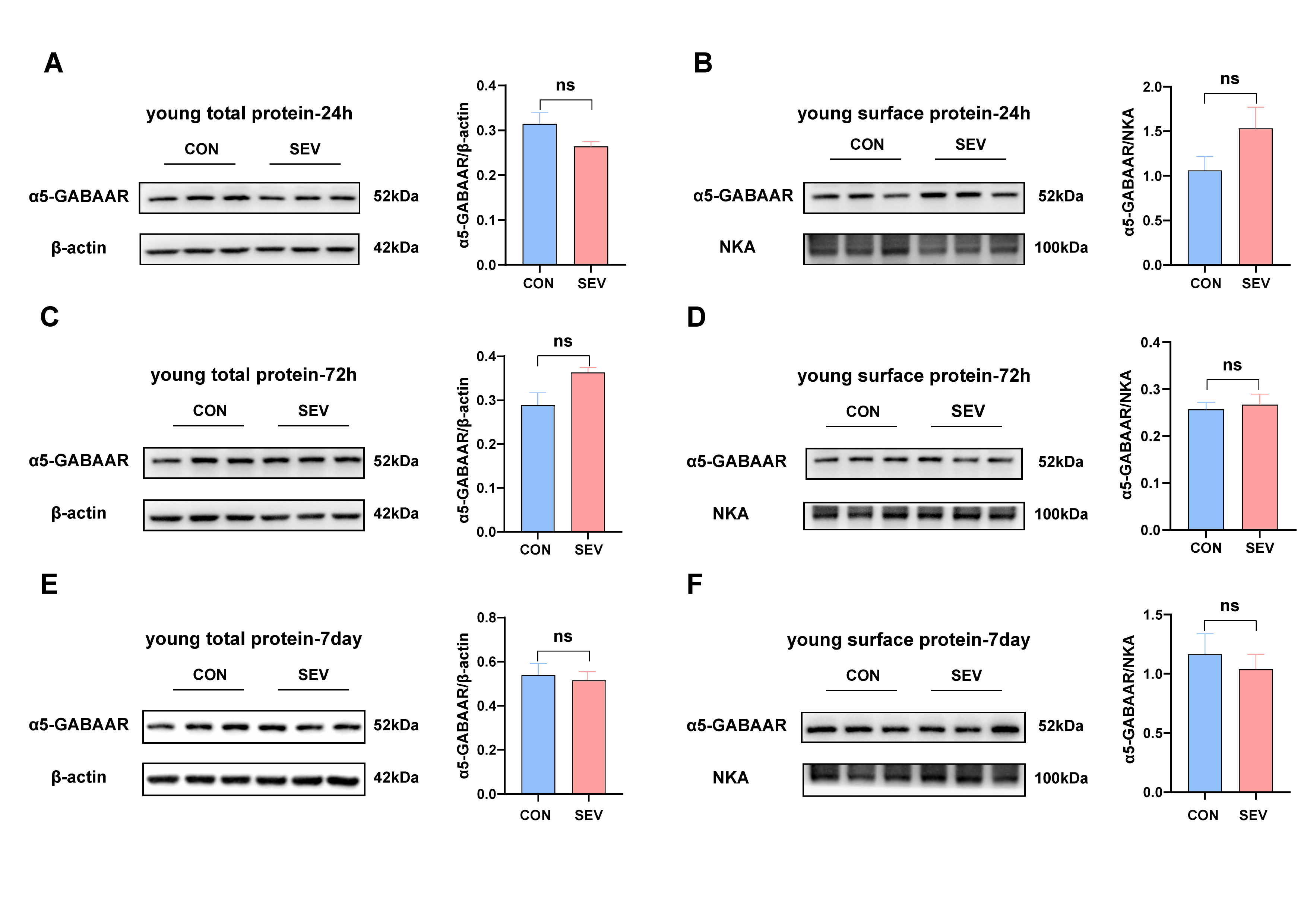

Supplement: Supplementary file 3 — Figure S3. [file ACEL-23-e14209-s001.zip › acel14209-sup-0003-FigureS3.tif]
