# Supplementary material for: Sevoflurane‐induced overexpression of extrasynaptic α5‐GABAAR via the RhoA/ROCK2 pathway impairs cognitive function in aged mice
Source: Aging Cell. 2024 Jun 2;23(9):e14209. doi: 10.1111/acel.14209 (PMC11488297; doi:10.1111/acel.14209)
Supplement: Supplementary file 2 — Figure S2. [file ACEL-23-e14209-s003.zip › FigureS2Caption.docx]

Figure S2. Sevoflurane has no effect on cognitive function in young mice. A-B. Freezing time of young mice during the tone-related test and contextual test (n = 7). C. Escape latency in the training stage (n = 7). D-F. Swimming speed, number of platform crossings, and length of time in the target quadrant in the probe test (n = 7). G. Representative traces in the Morris water maze. The escape latency was analyzed by two-way ANOVA with repeated measures followed by Tukey's analysis. All other data were analyzed using an unpaired Student's *t*-test. The data are presented as the mean ± SEM.
