# Supplementary material for: Sevoflurane‐induced overexpression of extrasynaptic α5‐GABAAR via the RhoA/ROCK2 pathway impairs cognitive function in aged mice
Source: Aging Cell. 2024 Jun 2;23(9):e14209. doi: 10.1111/acel.14209 (PMC11488297; doi:10.1111/acel.14209)
Supplement: Supplementary file 3 — Figure S3. [file ACEL-23-e14209-s001.zip › FigureS3Caption.docx]

Figure S3. Sevoflurane has no effect on the expression of α5-GABA_A_R in young mice. A, C, E. The expression of α5-GABA_A_R in hippocampal total protein samples in young mice (n = 3 per group). B, D, F. The expression of α5-GABA_A_R in hippocampal membrane protein samples in young mice (n = 3 per group). All the data were analyzed using an unpaired Student's *t*-test. * *P* < 0.05, ** *P* < 0.01. NKA, sodium potassium ATPase.
